# Automatic gait events detection with inertial measurement units: healthy subjects and moderate to severe impaired patients

**DOI:** 10.1186/s12984-024-01405-x

**Published:** 2024-06-18

**Authors:** Cyril Voisard, Nicolas de l’Escalopier, Damien Ricard, Laurent Oudre

**Affiliations:** 1 Université Paris Saclay, Université Paris Cité, ENS Paris Saclay, CNRS, SSA, INSERM, Centre Borelli, Gif-sur-Yvette, France; 2 Université Paris Cité, Université Paris Saclay, ENS Paris Saclay, CNRS, SSA, INSERM, Centre Borelli, Paris, France; 3 Service de Neurologie, Service de Santé des Armées, HIA Percy, Clamart, France; 4 Service de Chirurgie Orthopédique, Traumatologique et Réparatrice des Membres, Service de Santé des Armées, HIA Percy, Clamart, France; 5 Ecole du Val-de-Grâce, Service de Santé des Armées, Paris, France

**Keywords:** Pathological gaits, Gait analysis, Step detection, Pattern recognition, Intertial measurement units, Dynamic time warping

## Abstract

**Background:**

Recently, the use of inertial measurement units (IMUs) in quantitative gait analysis has been widely developed in clinical practice. Numerous methods have been developed for the automatic detection of gait events (GEs). While many of them have achieved high levels of efficiency in healthy subjects, detecting GEs in highly degraded gait from moderate to severely impaired patients remains a challenge. In this paper, we aim to present a method for improving GE detection from IMU recordings in such cases.

**Methods:**

We recorded 10-meter gait IMU signals from 13 healthy subjects, 29 patients with multiple sclerosis, and 21 patients with post-stroke equino varus foot. An instrumented mat was used as the gold standard. Our method detects GEs from filtered acceleration free from gravity and gyration signals. Firstly, we use autocorrelation and pattern detection techniques to identify a reference stride pattern. Next, we apply multiparametric Dynamic Time Warping to annotate this pattern from a model stride, in order to detect all GEs in the signal.

**Results:**

We analyzed 16,819 GEs recorded from healthy subjects and achieved an F1-score of 100%, with a median absolute error of 8 ms (IQR [3–13] ms). In multiple sclerosis and equino varus foot cohorts, we analyzed 6067 and 8951 GEs, respectively, with F1-scores of 99.4% and 96.3%, and median absolute errors of 18 ms (IQR [8–39] ms) and 26 ms (IQR [12–50] ms).

**Conclusions:**

Our results are consistent with the state of the art for healthy subjects and demonstrate a good accuracy in GEs detection for pathological patients. Therefore, our proposed method provides an efficient way to detect GEs from IMU signals, even in degraded gaits. However, it should be evaluated in each cohort before being used to ensure its reliability.

**Supplementary Information:**

The online version contains supplementary material available at 10.1186/s12984-024-01405-x.

## Background

The study of gait in medicine is an essential tool for evaluating the health and progression of patients with various diseases [1]. In this context, quantitative gait analysis can be used to finely evaluate the patient’s functional abilities, track the progression of their disease, measure the effectiveness of treatments, and develop personalized rehabilitation plans [2, 3].

In recent years, inertial measurement units (IMUs) have become widely developed in gait analysis due to their compact size, low cost, and ease of integration [4, 5]. They allow for objective and quantitative gait analysis, easy to use in healthy subjects, athletes [6] and patients, for example with neurological or orthopedic diseases [7]. They allow therefore the measurement of indicators on the gait semiology of patients, such as speed, stride length, double-support time, and balance [8]. Some of these features depend on the segmentation of strides and steps. Based on the historical description of gait in healthy subjects, 4 GE occur in a stride, in this order: Heel-Off (HO), Toe-Off (TO), Heel-Strike (HS), and Foot-Flat (FF) [9, 10]. Two main phases are described: the Stance Phase (StP) when the foot is on the ground between HS and TO, and the Swing Phase (SwP) when the foot is in the air between TO and HS [11, 12].

To accurately identify GEs from IMU signals, many techniques have therefore been developed. Currently, automatic detection of GEs on IMU recordings in healthy subjects has achieved a high degree of accuracy and continues to improve, with many efficient algorithmic techniques detecting GEs with a median absolute error of less than a tenth of a second [13–15]. However, the results on pathological subjects are often less precise, especially when gait is severely degraded. Due to the complexity of the sensory and motor commands that control gait, patients with advanced neurological pathologies can have particularly unstructured gaits [16–18]. For example, Ji et al. showed a 4 times lower accuracy in detecting the end of the step in hemiplegic subjects compared to healthy subjects [19]. Moreover, most of the algorithms tested on impaired patients have only been tested in a few specific diseases [20–23]. Therefore, one of the current challenges is to improve GE detection in pathological gaits.

A recent literature review has referenced the most commonly used mathematical principles in the exercise of IMU-based gait analysis [24]. This study summarizes research practices regarding IMU positioning, algorithmic methods, and algorithm validation processes. Over the past few years, the 3 most commonly found types of methods have been the Hidden Markov Model (HMM) [25], the Wavelet Transform (WT) [9], inspired by the study of ECGs, and rule-based detections (RBD) utilizing various mathematical tools [13, 26]. The study recommends the use of RBD with IMUs placed on the ankle or foot, validated using pressure sensors as ground truth. Recently, Deep Learning algorithms have also proven successful in detecting GEs [23].

Among the RBD algorithms, template-based methods have shown promising results in segmenting GEs in healthy subjects [10, 27, 28]. This technique relies on the creation of a reference dictionary of steps, which is used for segmentation of the signal by pattern recognition and extraction [29]. The use of a unique step dictionary allows for GE segmentation in healthy subjects [10, 29]. However, in neurological diseases, this method requires the addition of a solution for imprinting the patient’s step, such as an instrumented mat [28], making the deployment in routine clinical practice impossible. Another area for improvement concerns the use of different pattern extraction methods, such as Dynamic Time Warping (DTW) [10, 30]. DTW is a technique that promises precise detection of signal variations and similarity, and has already been used in gait analysis [27, 31].

The objective of this study is to develop a reliable algorithm for detecting degraded gaits using only IMUs, making it suitable for routine clinical use. To achieve this, we propose a new method for detecting Gait Events (GEs) in degraded gaits using IMUs. This method combines mathematical tools such as autocorrelation [32], the matrix profile algorithm [33] and the multiparametric Dynamic Time Warping (mDTW) algorithm [10].

This new automatic GE detection algorithm is based on the raw acceleration and gyration data collected by the IMUs and requires neither manual annotation nor external tools. To evaluate its efficiency, we tested it in comparison with an instrumented mat, considered the gold standard in gait segmentation [34], as well as with template-based state-of-the-art techniques [10, 29].

## Methods

### Cohorts

All the gait data were recorded at Percy Military Hospital (Clamart, France) from June 2018 to September 2021. 13 healthy control subjects (CS) who reported no medical impairment and were considered healthy after a clinical examination by medical doctors among investigators, 29 patients with multiple sclerosis (MS), and 21 post-stroke hemiplegic patients with spastic equino varus foot (EVF) requiring surgical intervention were enrolled. Included participants had to be mobile and able to walk 4 sets of 20 m with u-turn with a 3-minute break between each set, with or without medical assistance. Patients who were unable to walk, had a history of ankle or foot surgery, or had a history that could alter gait were excluded. Each subject in the CS cohort participated in between 1 and 3 sessions, each consisting of 4 to 42 experiments. Patients in the MS cohort participated in 1 or 2 sessions with 4 to 6 experiments each, as described in [28]. Patients in the EVF cohort were recorded preoperatively and during follow-up visits at 3 and 6 months. All participants provided written informed consent before inclusion. The study protocol followed the principles of the Declaration of Helsinki and was approved by the ethics committee “Protection of Persons North West III” (RCB ID: 2017-A01538-45).

### Data acquisition

#### Equipment

**Fig. 1:**
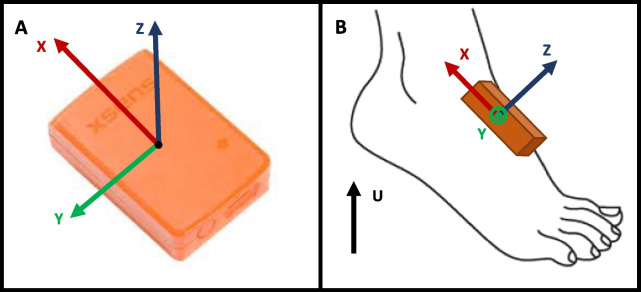
Presentation of the sensor. **A** Tw Awinda XSens^®^ sensor. **B** Definition of the axis for the sensor on the left foot

Two sensors MTw Awinda XSens^®^ (weight 16 g, dimensions 47 mm $\times$ 30 mm $\times$ 13 mm, sensitivity ± 2000 deg/s and ± 160 m/s2, XSens^®^ Technologies, Enschede, the Netherlands) (XS) were placed on the dorsal part of each foot of participants using Velcro bands. The sensor reference frame axes, which are used in subsequent analyses, are specified in Fig. 1. Apart from the axes, which must be correctly oriented, the exact position of the sensors is not measured, which is not required for the algorithm. A 6-meter GAITRite^®^ walkway (GR) was placed in a wide corridor of the hospital. The acquisition frequencies for XS and GR were 100 Hz and 120 Hz, respectively. Both systems were time-synchronized with the Awinda Recording and Docking Station to within 10 $\upmu$s.

#### Experience

**Fig. 2:**
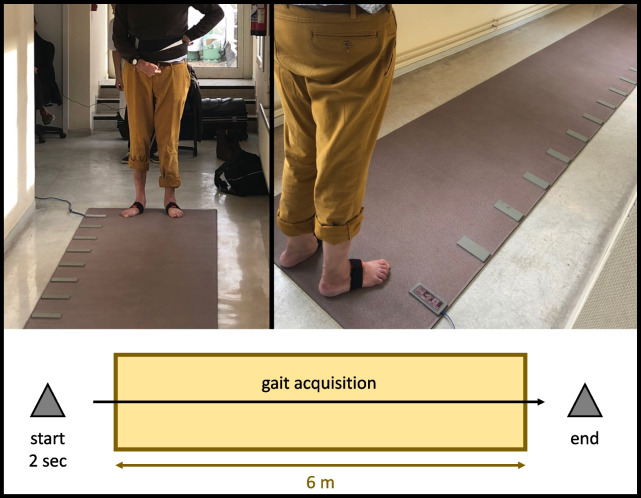
Gait recording protocol. The patient is equipped with two sensors Mtw Awinda XSens^®^ placed on the dorsal part of each foot using Velcro bands. Walking is initiated upstream of the active surface of the GR

An experiment involves walking straight for 10 ms at a comfortable speed while wearing the foot sensors, with 6 ms of the walk on the GAITRite® walkway. Initialization and termination of the walk are performed outside the mat, and the experiment can be conducted with or without footwear. In some cases, patients may use a gait aid such as a tripod cane, simple cane, or foot lift. The instructions given are consistent at the beginning of each test. Figure 2 illustrates the gait environment and provides a detailed description of the protocol.

#### Exporting and processing data

For XS recordings, the software XSens MVN studio software (MVN Studio, XSens, the Netherlands) is used to export the nine-dimensional signal (3D accelerations, 3D angular velocities, and 3D magnetic fields). To improve the quality of the signal, a low-pass Butterworth filter of order 8 with a cut-off frequency of 14Hz is applied. This filter setting is consistent with the trends reported in the literature [35].

For GR recordings, the GAITRite^®^ processing software is utilized to obtain the initial contact (IC) and final contact (FC) data. If the quality of the walk permits, automatic segmentation is performed. In cases where manual assistance is required, an expert is consulted. In either case, a visual inspection is conducted by the expert from GR signals and videos if available. The expert has the possibility to reject an event if its annotation is deemed “obviously incorrect”: an event in the middle of a phase without gyration or GR sensors turned on away from the footprint. Criteria for the exclusion of an experience or stride annotated by the GR are predetermined and outlined below:

- Logistical criteria that can invalidate an experiment: a sensor being misplaced or not recording for more than 0.5 s during the experiment, inability to export or corruption of a data file, or the subject’s inability to complete the entire mat crossing.
- Qualitative criteria that invalidate a GR event: an event partially outside the mat, an event detected too close to the end of the recording (duration less than the average duration of a stride), or obvious visual errors identified by the expert.

#### Data of interest

Based on the axes shown in Fig. 1, our algorithm focuses on two pre-processed signals. The first signal is the absolute norm of the total jerk free from gravity, which is obtained from the values of acceleration free from gravity:

$$\begin{aligned} \left\| \mathbf {j_{tot}} \right\| =\sqrt{\left( \frac{dfreeAcc_{x}}{dt} \right) ^{2} + \left( \frac{dfreeAcc_{y}}{dt} \right) ^{2} + \left( \frac{dfreeAcc_{z}}{dt} \right) ^{2}}. \end{aligned}$$

The second signal is the gyration in the sagittal plane directly measured by the sensor: $\omega _{y}$.

We treat both signals, which are sampled at 100 Hz by the IMUs, as time series. If data is missing, we complete it using quadratic interpolation. The jerk time series and the gyration in the sagittal plane are respectively designed as $\textbf{J}$ and $\mathbf {\Omega }$ in the following analysis.

### Algorithm procedure

**Fig. 3:**
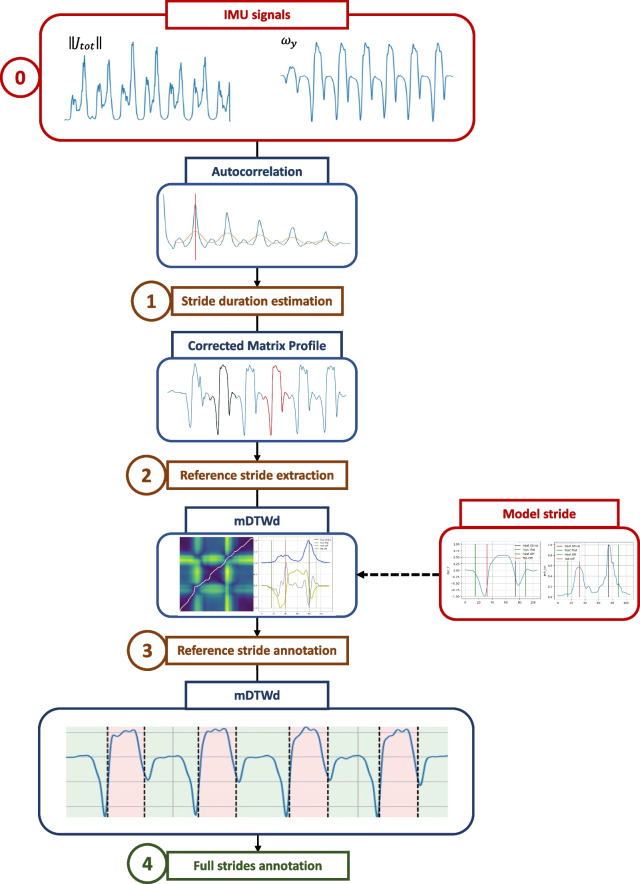
Flowchart of the GE detection method. Schematic representation of the 4 parts of the algorithm. The color of the boxes is: red for input data, blue for the corresponding tools and illustrations, brown for intermediate steps, and green for the output result

The algorithm is built in 4 parts, and an illustration of the whole process is proposed in Fig. 3.

Firstly, we estimate stride length using the autocorrelation method [32]. This enables us to identify a reference stride by comparing it to a pre-defined model stride for similarity using a targeted matrix profile algorithm [33]. Next, we use the jerk and gyration signals and apply a multiparametric Dynamic Time Warping (mDTW) algorithm to annotate the reference stride. Finally, we use correlation and mDTW to detect all GEs of the signal using the annotated stride as a template, following the same method as previously employed [10]. All these steps are fully automatic and are detailed below.

#### First part: stride duration inference with autocorrelation

This section aims to estimate a consistent value for the average duration of a stride. Autocorrelation is a commonly used signal-processing technique that involves cross-correlating a signal with itself. It can be used to estimate the period of an imperfect periodic signal that does not contain a single dominant frequency [36].

**Fig. 4:**
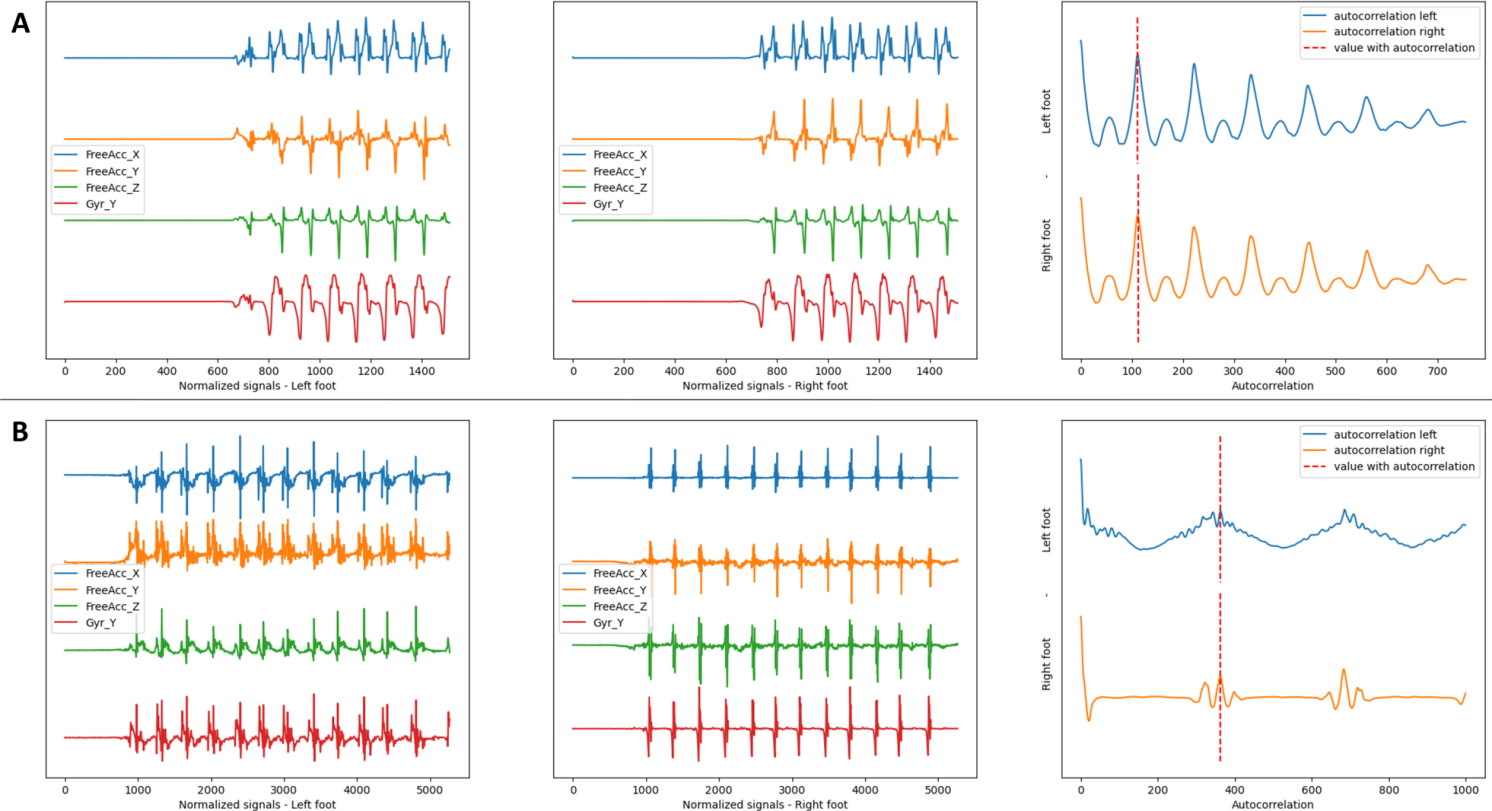
Autocorrelation signal. **A** Control subject. **B** Patient from the EVF cohort. The two graphs on the left show the preprocessed signals of interest for both feet. The righthand graph is the resulting multiparametric autocorrelation for each foot and gives the estimated return value of the duration of a stride (dot line)

We considered the free accelerations and gyrations recorded by foot-level IMUs to be stationary and periodic depending on the regularity of the subject. We computed the autocorrelation for $\mathbf {\Omega }$ and $\textbf{J}$ components. The estimated stride duration was determined by matching the first autocorrelation peak. We assumed that the expected values of the average stride duration were the same for each foot. To avoid overestimation, we chose the shorter of the two stride estimates. This assumption does not mean that the steps have the same duration and therefore does not erase a potential asymmetry. An illustration of the signals and the peak detection process is provided in Fig. 4.

#### Second part: reference stride isolation with matrix profile

Once the average duration *L* of a stride is estimated, we want to find a reference stride. Therefore, the most recurrent pattern of *L*-size must be extracted from the signal. Matrix Profile (MP) is a recent reference [37, 38] efficient for the pattern extraction task. This time series processing technique uses normalized Euclidean distance to calculate the similarity between subsequences of the original sequence. The resulting output highlights repeating patterns in the data sequence.

To adapt and personalize the pattern detection, so as the start and the end of the pattern corresponds to phenomena of interest, Dau et al. [39] proposed to add an annotation vector (AV). AV is a time series which is explicitly describing the preferred location or expected behavior of the pattern, thus limiting the risk of detecting insignificant patterns [40]. A low value indicates that the subsequence starting at this index is not a valuable pattern, and should therefore be rejected. Conversely, higher values mean that the subsequence at that location should be favored for pattern detection. The combination of AV and MP leads to a corrected matrix profile (CMP).

In our algorithm, we computed a CMP from $\mathbf {\Omega }$ and a dimensionless AV that favors locations in order to have large variations for $\textbf{J}$ and $\mathbf {\Omega }$ in the center of the search window. Our objective was to center the SwP. AV was defined as follows:

1 $$\begin{aligned} AV_{i}=\sum _{k=i + \frac{m}{3}}^{i+\frac{2m}{3}} \frac{\omega _{k}}{\omega _{max}} + \frac{j_{k}}{j_{max}}. \end{aligned}$$

**Fig. 5:**
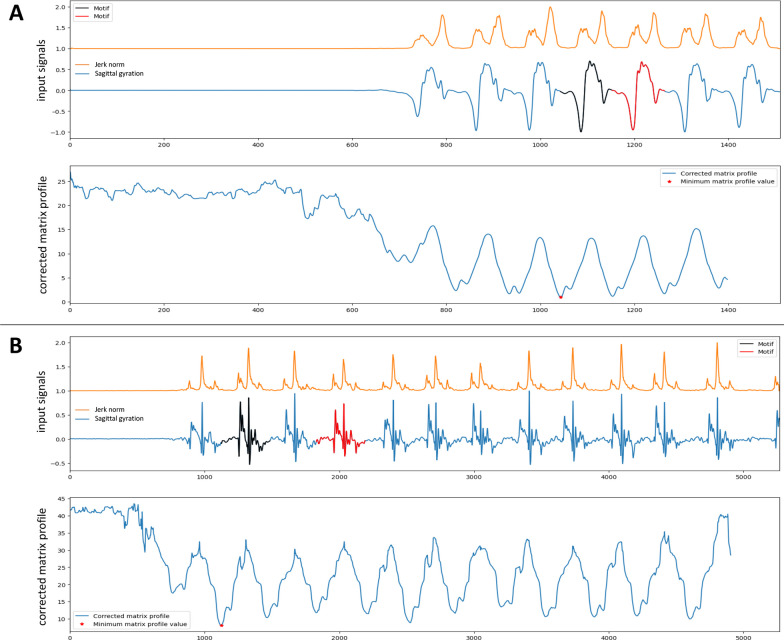
Corrected matrix profile. **A** Control subject. **B** Patient from the EVF cohort. Top: gyration signal (blue) and jerk signal (orange). Bottom: CMP, the red star indicates the minimum value of the CMP and allows the detection of the pattern (black) and its nearest neighbor (red)

An illustration of pattern recognition by CMP using the example of gyration is shown in Fig. 5.

#### Third part: reference stride annotation with DTW

The pattern isolated in the previous section corresponds to a complete stride, probably centered on the SwP. The next step is to annotate this reference stride with the best estimates of TO and HS. To do this, a model stride was used to annotate each reference stride using DTW.

**Fig. 6:**
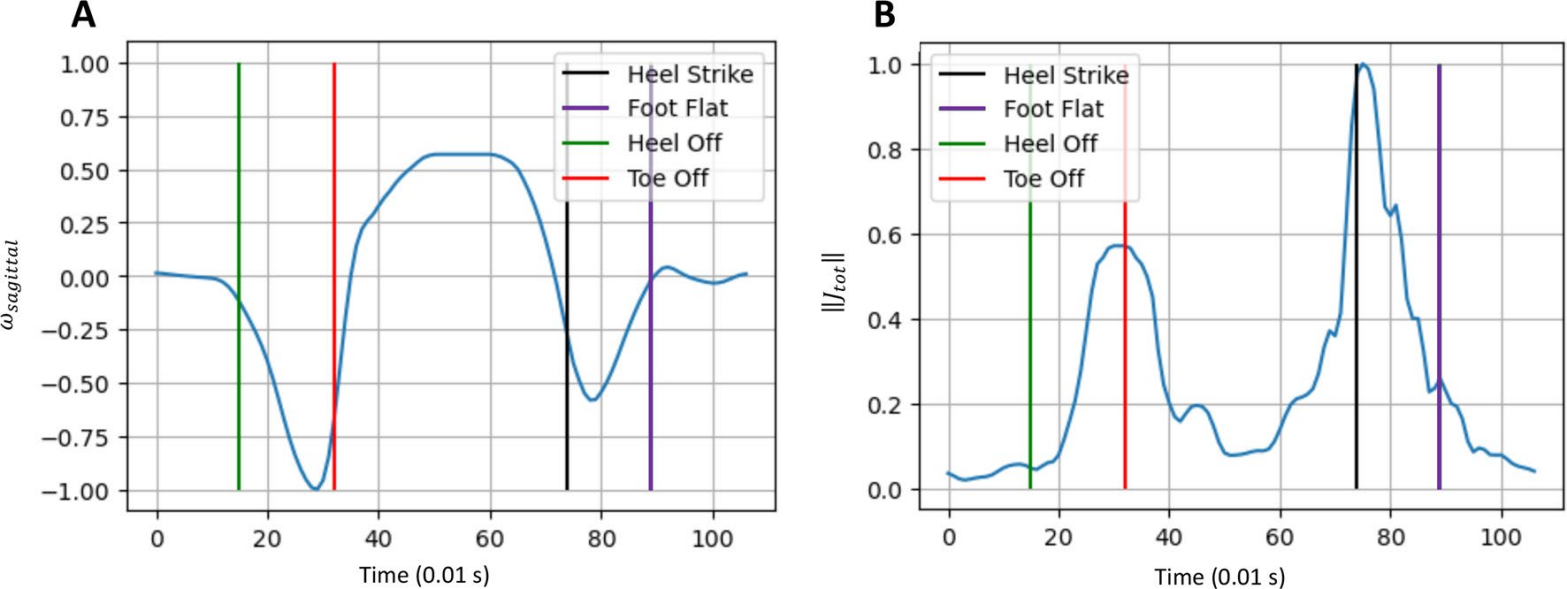
Model stride. Stride from a healthy subject used as a model for all detections. **A** Gyration (blue) in the sagittal plane. **B** Total jerk (blue). For each figure, the 4 events of the stride are represented. TO and HS were given by the GR. FF and HO were visually estimated and given as an indication

*Model stride*. We extracted a “model stride” from our dataset of healthy subjects, which has all 4 stride events annotated. Specifically, TO and HS were annotated with the GR, while FC and IC were annotated with their respective events. FF and HO were estimated and are provided as an indication. The signals of interest corresponding to this stride are illustrated in Fig. 6.

*DTW*. The Dynamic Time Warping (DTW) algorithm is a distance measure that aligns and transforms two time series through a non-linear transformation to obtain an optimal match between them. Its objective is to determine a measure of their similarity and obtain a matching path between the points called the “warping path”. We opted for a multidimensional DTW (mDTW) taking into account both time series $\textbf{J}$ and $\mathbf {\Omega }$. Following the recommendations of [41], we used a mDTW with dependence between signals, called mDTWd, using normalized Euclidean distance with normalized signals. Because of the probable presence of the SwP in the middle of the reference stride, we used an additional constraint with an Itakura parallelogram with radius *r *= 2 [42].

**Fig. 7:**
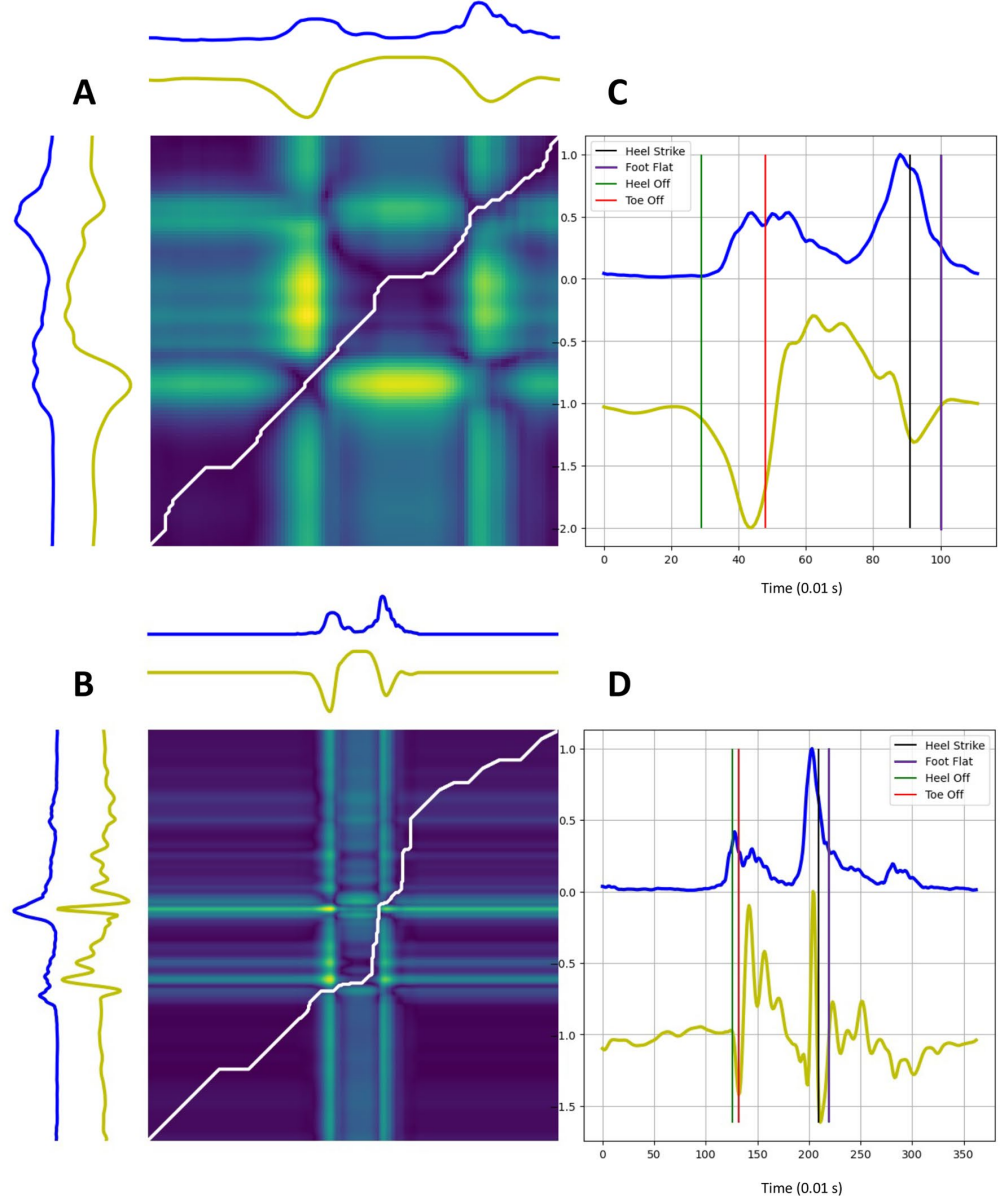
Annotated reference stride. Top: healthy subject from the CS cohort. Bottom: patient from the EVF cohort. **A**, **B** mDTWd matrix between model stride (up) and reference stride (left) signals with the corresponding warping path (white line). **C**, **D** Annotation of the reference with the 4 GEs. Blue line: jerk. Yellow line: gyration

*Reference stride annotation*. To compare and annotate the reference stride with the model stride, we first shifted the model stride to ensure that the SwP was in the same position in both series. We then applied mDTWd to find the optimized path under constraints. Finally, we used this warping path to match the events of both series. We assumed that the last point of the subject’s series that matched with the TO point of the model corresponded to the TO estimation, and the first point of the subject’s series that matched with the HS point of the model corresponded to the HS estimation. Figure 7 illustrates the annotation protocol and an example of the result.

#### Fourth part: segment the entire walk with a template-based detection

The annotated stride obtained at the end of the previous section is used as a reference for the detection of GE throughout the signal using the template-based method. The method used is based on the one described in the article by Dot et al. [10], which uses mDTW on the series while adapting the parameters due to degraded strides: $\lambda$ = 0.4 and $\mu$ = 0.1. At the end of this final part, all the detected strides should be completely annotated with the best findings of TO and HS, as shown in 8.

**Fig. 8:**
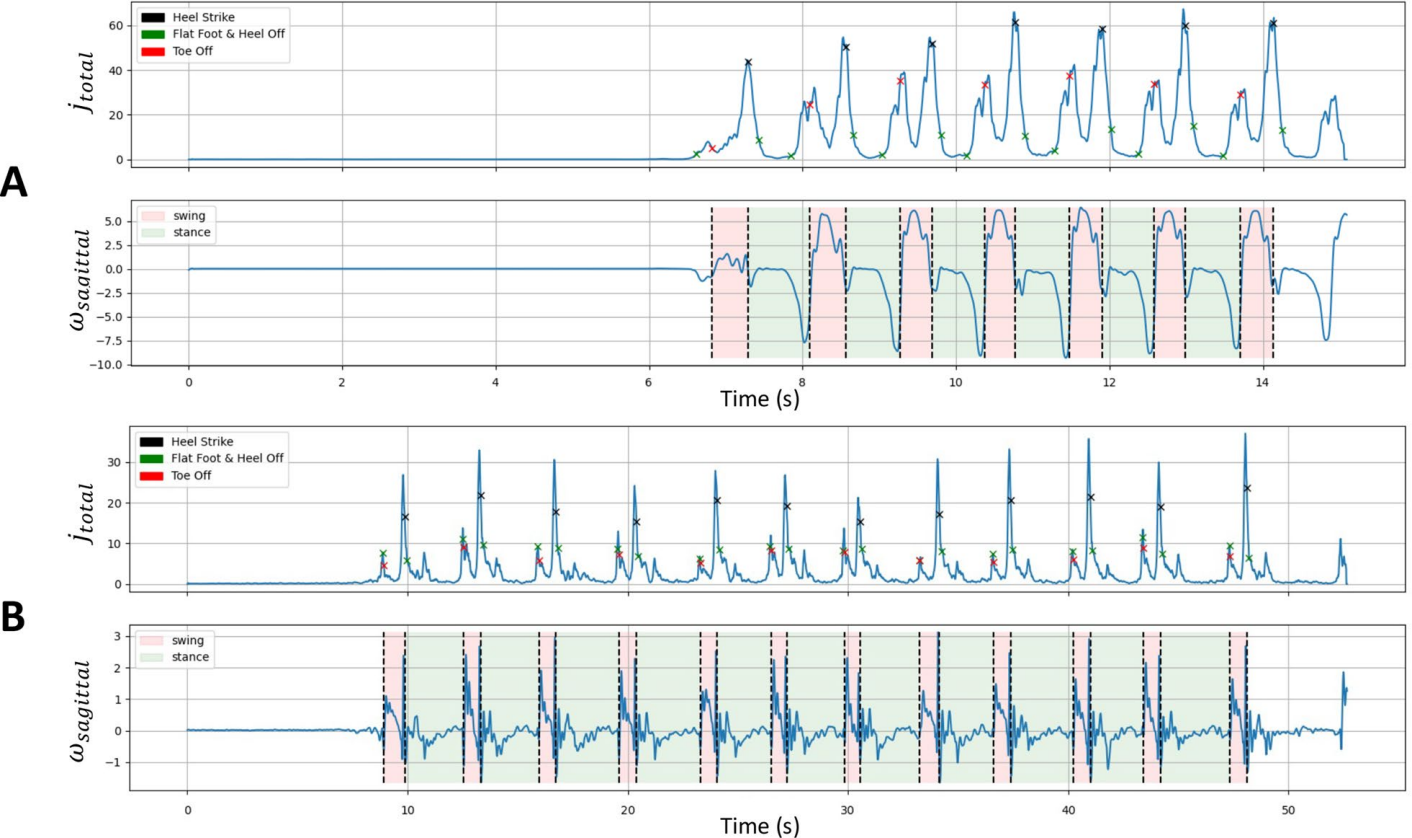
Final gait segmentation. **A** Healthy subject from the CS cohort. **B** Patient from the EVF cohort. GEs detected by the algorithm were reported on the jerk signal (*top*). Gait phases deducted from GEs were reported on the gyration signal (*bottom*)

### Technical precisions

The algorithm was implemented with python 3.8, on the PyCharm 2022.3 IDE. In part 2, we used the *compute* function from the Matrix Profile Foundation’s python package, with the default parameters. In parts 3 and 4, we used the python *tslearn* package [43] to compute mDTWd.

### Performance analysis

The accuracy of the proposed method is assessed by comparing the correctly detected times for HS and TO to the annotations IC and FC of the GR gold standard. The following metrics were used:

- Events. As described before, the GEs of interest in the study are TO and HS.
- Annotated events. These are the events IC and FC annotated by the GR, considered the Gold Standard.
- Detected events. These are the events HS and TO detected by the algorithm between the beginning and the end of the gait experience on the mat.
- Recall (sensitivity). This is the proportion of events annotated by the GR that have been correctly detected by the algorithm. An annotated event is considered correctly detected if the duration between this annotated event and the nearest detected event of the same nature (TO for FC; HS for IC) does not exceed 20% of the duration of a stride. A detected event can be used only one time. Sensitivity is defined as the ratio between the number of correctly detected annotated events and the total number of annotated events.
- Precision. This is the proportion of detected events that effectively correspond to an annotated event. A detected event is considered to be correct if the duration between this detected event and the nearest annotated event of the same nature (FC for TO; IC for HS) does not exceed 20% of the duration of a stride. An annotated event can be used only one time. Precision is defined as the ratio between the number of correctly detected events and the total number of detected events.
- F1-score. This is the harmonic mean of precision and recall.
  2 $$\begin{aligned} F1_{score}= 2 \times \frac{recall * precision}{recall + precision}. \end{aligned}$$
- $\Delta$TO. For a correctly detected TO event, it is the absolute difference in time (ms) between the corresponding annotated FC and the detected TO.
- $\Delta$HS. For a correctly detected HS event, this is the absolute difference in time (ms) between the corresponding annotated IC and the detected HS.
- Mean $\Delta$HS and Mean $\Delta$TO. For each type of event, it is the average of the measured absolute differences for the correctly detected and annotated events.

This performance was then evaluated against the performance of the algorithm of Dot et al. [10].

### Statistical analysis

The main performance metric used to evaluate the method was the F1-score, which is a combined measure of recall and precision. An F1-score of less than or equal to 0.95 was considered indicative of low performance. The second outcome measure was the median absolute error in GE detection compared to the gold standard, which reflects accuracy. The method was compared to the state of the art. For non-normally distributed data, such as $\Delta$HS and Mean $\Delta$TO, medians and interquartile ranges (IQRs) were reported, and the Wilcoxon-Mann–Whitney test (WMW-test) was used to evaluate the significance of the results. Differences were represented as relative values for visualization. The normality of the data was assessed with the Shapiro-Wilk test. For normally distributed data, means and standard deviations (SDs) were reported. All statistical analyses were computed with R Studio 2023.03.0-386.

## Results

### Participants

We included 13 CS individuals, 29 MS patients, and 21 EVF patients. The baseline characteristics of the subjects and patients are filled in Table 1.

**Table 1:** Baseline characteristics of each cohort

	CS (n = 13)	MS (n = 29)	EVF (n = 21)
Sex (M/F)	6/7	13/16	9/12
Age (years)	26.6 (2.0)	59.2 (10.0)	54.0 (14.8)
Height (m)	1.69 (0.09)	1.69 (9.7)	1.68 (10.9)
Weight (kg)	63.3 (14.8)	67.5 (17.6)	71.2 (14.9)
EDSS	–	6 [2–7]	–

For age, height and weight, mean and standard deviations are given. Median and extreme values of EDSS are given for MS patients. There is no validated severity score for the EVF cohort, but it should be noted that the surgical indication for the equine varus foot is given in the most severe cases

* CS* control subjects,* MS* multiple sclerosis patients,* EVF* equino varus foot patients

**Fig. 9:**
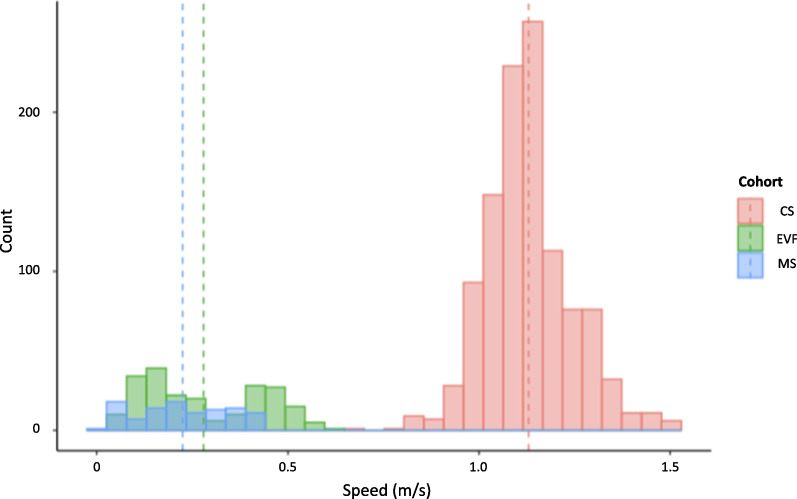
Walking speed distribution for each cohort. Dot lines represent mean values

To provide an overview of the quality of the data and gait characteristics, Table 2 presents the number of steps and the mean walking speed for each cohort. Figure 9 illustrates the distribution of walking speed within each cohort.

**Table 2:** Gait parameters

	CS (n = 13)	MS (n = 29)	EVF (n = 21)
Speed in m/s (SD)	1.13 (0.11)	0.23 (0.12)	0.28 (0.15)
Total steps	8410	3442	4180
Steps per exp (IQR)	8 (7–8)	27 (22–36)	17 (13–22)

Number of steps, mean (SD) of gait speed, and median (IQR) number of steps per experience

*CS* control subjects,* MS* multiple sclerosis patients,* EVF* equino varus foot patients

**Fig. 10:**
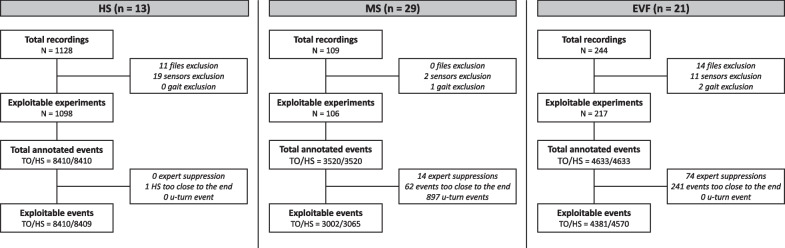
Flowchart of the data collected and analyzed in the study. Rules for deleting and correcting data are provided in Methods

Figure 10 provides a detailed overview of the recorded dataset that is utilized in the subsequent analyses (rules are specified in the Methods section).

### Performance and accuracy of the detection method compared to gold-standard

The results of the detailed algorithm are presented in Table 3. For the CS cohort, the F1-score was 100%, indicating perfect and exact detection of all strides. Among the well-detected annotated strides, the median $\Delta$TO was 8 ms (IQR [3–15] ms), and the median $\Delta$HS was 7 ms (IQR [3–12] ms). For the MS cohort, the F1-score was 99.4%, with a recall of 99.7% and a precision of 99.2%. The median $\Delta$TO was 23 ms (IQR [10–50] ms), and the median $\Delta$HS was 15 ms (IQR [7–30] ms). For the EVF cohort, the F1-score was 96.3%, with a recall of 96.7% and a precision of 96.0%. The median $\Delta$TO was 27 ms (IQR [15–48] ms), and the median $\Delta$HS was 25 ms (IQR [10–50] ms).

**Table 3:** $\Delta$TO and $\Delta$HS of our main algorithm for each cohort

	CS (n = 16819)	MS (n = 6067)	EVF (n = 8951)
F1	100	99.4	96.3
Recall (%)	100	99.7	96.7
Precision (%)	100	99.2	96.0
TO events	8410	3002	4381
Median $\Delta$TO [IQR]	8 [3–15]	23 [10–50]	27 [15–48]
HS events	8409	3065	4570
Median $\Delta$HS [IQR]	7 [3–12]	15 [7–30]	25 [10–50]

All the results are given in milliseconds

*CS* control subjects,* MS* multiple sclerosis patients,* EVF* equino varus foot patients,* TO* Toe-Off,* HS* Heel-Strike

**Fig. 11:**
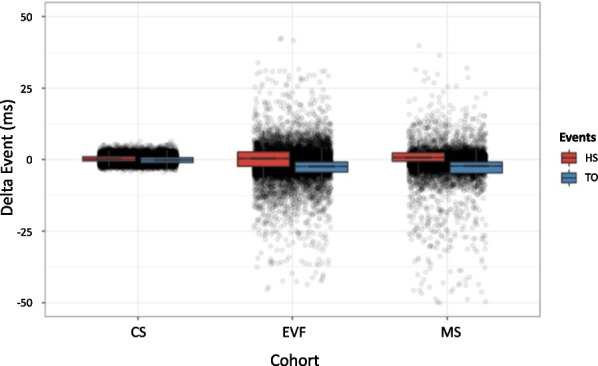
Boxplot of $\Delta$HS and $\Delta$TO. Each dot represents a correctly detected and annotated step

The boxplot of $\Delta$HS and $\Delta$TO for the correctly detected and annotated steps of each cohort are presented in Fig. 11. The details for each patient or subject included in the study are provided for illustrative purposes in the Supplementary materials [see Additional file 1].

**Fig. 12:**
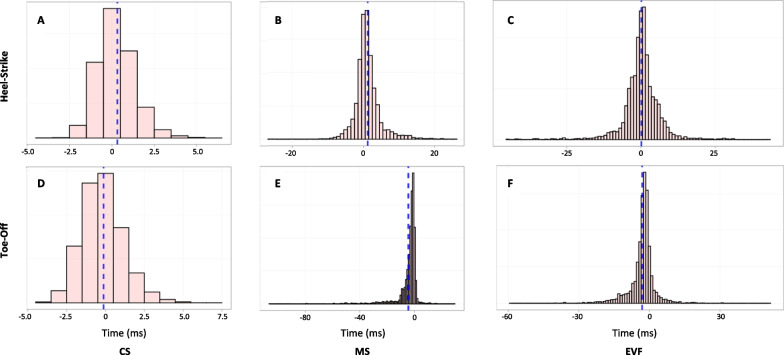
Histograms of $\Delta$HS (**A–C**) and $\Delta$TO(**D–F**) for each cohort. Dot lines represent the mean error

More detailed histograms are displayed in Fig. 12.

### Performance and accuracy of the detection method compared to state-of-the-art

The recently validated Dot [10] algorithm uses the same protocol and the same sensor position, which allows us to use it as a reference to test the results of our algorithm. We applied it to our data set. For CS, the F1-score was unchanged at 100%. The median absolute error for TO was 13 ms (IQRs [7–22] ms) and the median absolute error for HS was 10 ms (IQRs [5–18] ms), which was significantly poorer than our performance (p-value < 0.0001). For MS and EVF, the F1-scores were satisfactory (< 95%) and the median deviations for HS and TO were significantly worse than for our algorithm (p-value <0.0001). All the results and tests are reported in Table 4.

**Table 4:** $\Delta$TO and $\Delta$HS of state-of-the-art algorithm for each cohort

		CS		MS		EVF	
		Value	*p-value*	Value	*p-value*	Value	*p-value*
Dot 2020 [10]	F1-score (%)	100	–	99.1	–	95.7	–
	Recall (%)	100	–	99.4	–	96.1	–
	Precision (%)	100	–	98.8	–	95.4	–
	Mean $\Delta$TO [IQR]	13 [7–22]	< **0.0001**	15 [7–53]	< **0.0001**	20 [8-68]	< **0.0001**
	Mean $\Delta$HS [IQR]	10 [5–18]	< **0.0001**	23 [8–66]	< **0.0001**	43 [17-82]	< **0.0001**

All the results are given in milliseconds. When displayed, p-value is the result of the WMW-test with our full algorithm. F1-scores lower than 95% and p-values lower than 0.05 are displayed in bold

*CS* control subjects,* MS* multiple sclerosis patients,* EVF* equino varus foot patients,* TO* Toe-Off,* HS* Heel-Strike

### Ablation study

To assess the necessity of the mathematical tools used in parts 2– 4, we implemented alternative algorithms that either removed these tools or replaced them with simpler ones. We then compared the results obtained with our original algorithm using a WMW-test. We present here 3 of the alternative algorithms that were tested.

- Test 1: without Jerk signal. Only the gyration signal was considered for the whole protocol.
- Test 2: without MP. We replaced the pattern detection with the MP algorithm with a random selection of a portion of the signal of the same duration, located between the beginning and the end of the gait on GR to ensure that a walking period was selected.
- Test 3: without DTW. We replaced the DTW-based method used for annotating the reference stride in part 3 with a correlation optimization method. Similarly, for part 4, the stride detection on the entire signal was performed solely using correlation without any readjustment by DTW.

Table 5 summarizes the results obtained, clearly demonstrating the relative importance of each of the tools used.

**Table 5:** $\Delta$TO and $\Delta$HS of alternative algorithms for each cohort

		CS		MS		EVF	
		Value	*p-value*	Value	*p-value*	Value	*p-value*
Test 1	F1-score (%)	100	–	99.1	–	95.7	–
	Recall (%)	100	–	99.4	–	96.1	–
	Precision (%)	100	–	98.8	–	95.4	–
	Mean $\Delta$TO [IQR]	8 [3–15]	0.801	30 [13-77]	**< 0.0001**	31 [17–70]	**< 0.0001**
	Mean $\Delta$HS [IQR]	8 [3–18]	**< 0.0001**	19 [8-44]	**< 0.0001**	38 [15–80]	**< 0.0001**
Test 2	F1-score (%)	**56.4**	–	**49.2**	–	**57.2**	-
	Recall (%)	56.9	–	48.6	–	57.1	–
	Precision (%)	55.9	–	49.6	–	57.4	–
	Mean $\Delta$TO [IQR]	17 [7-71]	**< 0.0001**	50 [18-177]	**< 0.0001**	58 [23–177]	**< 0.0001**
	Mean $\Delta$HS [IQR]	13 [5-67]	**< 0.0001**	34 [10-166]	**< 0.0001**	60 [20–171]	**< 0.0001**
Test 3	F1-score (%)	97.9	–	98.2	–	95.2	–
	Recall (%)	97.9	–	98.6	–	95.5	–
	Precision (%)	97.9	–	98.0	–	94.9	–
	Mean $\Delta$TO [IQR]	33 [15–90]	**< 0.0001**	42 [18–90]	**< 0.0001**	57 [27–100]	**< 0.0001**
	Mean $\Delta$HS [IQR]	33 [13–67]	**< 0.0001**	32 [14–73]	**< 0.0001**	57 [25–107]	**< 0.0001**

All the results are given in milliseconds. When displayed, p-value is the result of the WMW-test with our full algorithm. F1-scores lower than 95% and p-values lower than 0.05 are displayed in bold

*CS* control subjects,* MS* multiple sclerosis patients,* EVF* equino varus foot patients,* TO* Toe-Off,* HS* Heel-Strike

## Discussion

Gait analysis using IMU has gained widespread popularity, and several algorithms have been developed and demonstrated their efficacy in detecting gait events (GEs) of healthy individuals [4, 13, 24, 44]. In clinical settings, measuring gait performance provides valuable additional information about walking activity and spontaneous gait characteristics, such as in the follow-up of neurological patients [5, 18, 45] or in preoperative examinations [46, 47]. While some teams use only trunk wearable sensors that provide global features such as smoothness [48], a more precise analysis requires GE detection, which can be done with two separate IMUs on each lower limb [47].

Our algorithm provides automated and reliable GE detection, which is effective not only for healthy subjects but also for pathological gaits (precision $\ge$ 95%, recall $\ge$ 95%, and F1-score $\ge$ 95%). Other previously published techniques have shown similar F1-scores (Table 6). Hidden-Markov-Model [25], wavelet transform [49], and some rule-based methods have been shown to be effective among these techniques.

**Table 6:** $\Delta$Start (IC or HS) and $\Delta$End (FC or TO) reported in the literature for healthy subjects and pathological gaits

Publication	Subjects	F1-score (%)	$\Delta$Start (ms)	$\Delta$End (ms)
Dot et al. 2020 [10]	Healthy	99	15 (18)	16 (13)
Flood et al. 2020 [50]	Healthy	99	16 (7)	40 (16)
Romijnders et al. 2021 [22]	Elderly	100	32	29
	Hemiparetic	100	31	40
	PD	100	33	40
Perez-Iribarra et al. 2020 [51]	Healthy	99	75 (40)	29 (24)
	Hemiparetic	97	68 (42)	52 (39)
	Myelopathic	96	58 (43)	54 (41)
Trojaniello et al. 2014 [26]	Elderly	100	10	20
	Hemiparetic	100	17	21
	PD	100	15	22
	Choreic	100	12	18
Ji et al. 2019 [49]	Healthy	$\ge$ 95	60	20
	Hemiparetic	$\ge$ 95	40	180
Vienne-Jumeau et al. 2020 [28]	Healthy	100	15 (7)	19 (9)
	WA pMS^a^	99	100 (60)	50 (40)
	NA pMS^b^	99	60 (70)	30 (40)
Storm et al. 2018 [52]	WA pMS^a^	–	60 (20)	100 (50)
	NA pMS^b^	–	70 (30)	100 (30)

^a^WA-pMS: pMS needing a walking aid

^b^NA-pMS: pMS not needing a walking aid

Regarding the median time error between detected events and GR annotated events, our method demonstrates results of below 10 ms in healthy subjects, which is consistent with the latest algorithms [22, 49, 50], including Deep Learning algorithms [23]. In MS and EVF pathological subjects, our results show moderate impairment and give acceptable results centered around 20 ms. However, most existing algorithms are flawed when analyzing pathological gaits in some neurological pathologies such as multiple sclerosis [28] and hemiplegic patients [47]. At last, some methods require training phases for each patient that may require additional equipment [28]. Although step detection seems to be performant for pediatric hemiplegic patients [53], it remains difficult for post-stroke hemiparetic patients [54].

Our algorithm is built on a Template-Based Approach that has already been successfully applied [29]. Until now, this approach required the implementation of step dictionaries that were not efficient enough for pathological gaits. Additionally, when these dictionaries were customized [28], they required the joint use of an instrumented mat as GR during the training phase, which compromised clinical deployment. To overcome this, our method extracts the template directly from each record. The results show that CMP described by VanBenschoten et al. [33] may be an important part in increasing the precision and accuracy of detection. The addition of DTW, often used in HMM, has also increased detection performance as it does not depend on the time factor but only on the similarity of the signals [30]. Dot et al. [10] showed that using a single template based on gyration signals and associated with non-linear deformations may be sufficient to model the gait of healthy subjects, and our algorithm is consistent with this result. However, whereas Dot et al. did not efficiently detect GEs in highly pathological gaits, our method maintains satisfactory results in neurological patients. As suggested by [31], the use of mDTWd has shown better efficiency.

Our study may have some limitations. Firstly, only short straight-line gait has been studied, whereas the u-turn may provide valuable information in monitoring neurological pathologies [55, 56]. Secondly, the experiments took place in a safe, hospital environment. Even if these two conditions are suitable for the examination of gait in daily clinical practice, they do not reflect the patient’s gait in a free environment and may have limitations when transposing the method to real-life gait recordings. Finally, the gold standard we used has limitations in analyzing highly pathological steps. The GR automated step detection is ineffective since it relies on a well-paced right-left alternation respecting a median line. Even the manual annotation of steps via the GR software with a video fails when patients do not have a classic HS-TO stride sequence. For instance, in the case of foot drop, the stride’s initial contact will not always be the HS but could be a “Toe-Strike.” Additionally, in some patients, the foot does not leave the ground due to an important motor deficit, yet the foot moves and no longer really carries the body’s load. Bruening et al. suggests using different algorithms depending on the gait pattern [57]. For us, it is essential that one reproducible algorithm allows step detection for all gait patterns, particularly for longitudinal follow-up of operated patients, as surgery may significantly alter their gait. Furthermore, this raises the question of the step’s definition. While some teams consider Initial Contact and Final Contact as genuine GEs, we prefer to consider HS and TO, which define step stability during the StP. Thus, for foot drop, Toe-Strike could be considered part of the SwP since the patient has not yet stabilized their foot on the ground. Considering these factors, template analysis by IMU complements contact analysis by instrumented mats or force platforms.

Another issue to consider is the localization of wearable sensors for gait analysis [9, 58]. Trojanello et al. and Romijnders et al. use a shank sensor, which may be more sensitive to the anterior-posterior acceleration movement during the static phase, for which the foot sensor does not provide any information [23, 26]. This location may be more suitable for detecting gait events. However, for the analysis of step width and asymmetry, the sensor located on the foot will be more sensitive to variations and thus provide more relevant information. It is therefore a compromise between detecting gait events and analyzing gait parameters. It could also be interesting to use all 4 sensors to combine their interests, even if this would make deployment of the measurement more complex.

## Conclusion

Our Adaptive Non-Linear Approach to Step Detection using IMU has proven to be effective in analyzing the gait of healthy individuals and some pathological gait, without the need for an annotated dictionary or the use of a gait recognizer. This innovative tool has the potential to enable real-time gait analysis of neurological patients and can be used as a routine clinical tool throughout their follow-up. Our next objective is to provide an instantaneous gait analysis that can be deployed in clinical routines.

### Supplementary Information

Supplementary Material 1.

## Data Availability

The datasets generated for this study are available on request to the corresponding author.

## Abbreviations

IMU: Inertial measurement units
GE: Gait event
HO: Heel-off
TO: Toe-off
HS: Heel-strike
FF: Foot-flat
StP: Stance phase
SwP: Swing phase
DTW: Dynamic time warping
mDTW: Multiparametric dynamic time warping
mDTWd: Multiparametric dynamic time warping with dependence
MP: Matrix profile
CMP: Corrected matrix profile
AV: Annotation vector
CS: Control subject
MS: Multiple sclerosis
EVF: Equino varus foot
XS: MTw Awinda XSens^®^
GR: GAITRite^®^ walkway
IC: Initial contact
FC: Final contact
IQR: Inter quartile range
SD: Standard deviation
