# Supplementary figures and images for: Automatic gait events detection with inertial measurement units: healthy subjects and moderate to severe impaired patients
Source: J Neuroeng Rehabil. 2024 Jun 18;21:104. doi: 10.1186/s12984-024-01405-x (PMC11184826; doi:10.1186/s12984-024-01405-x)

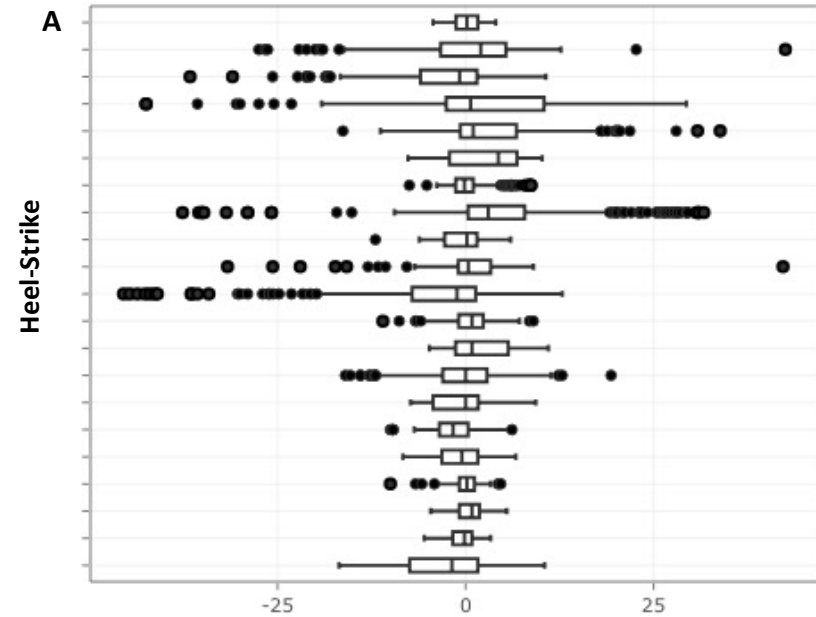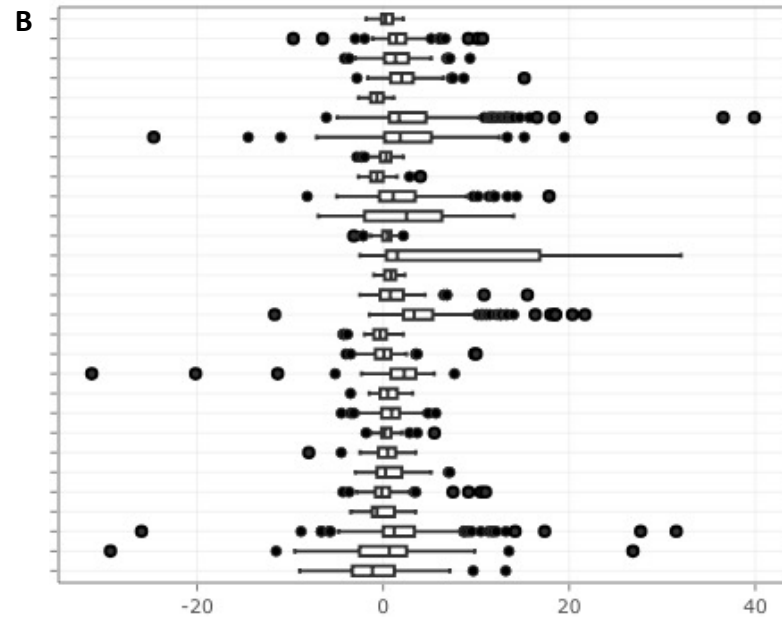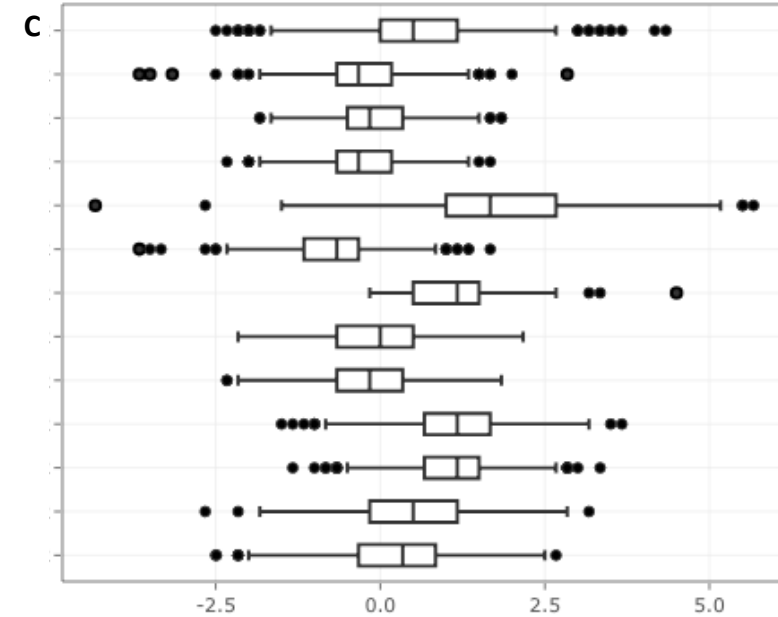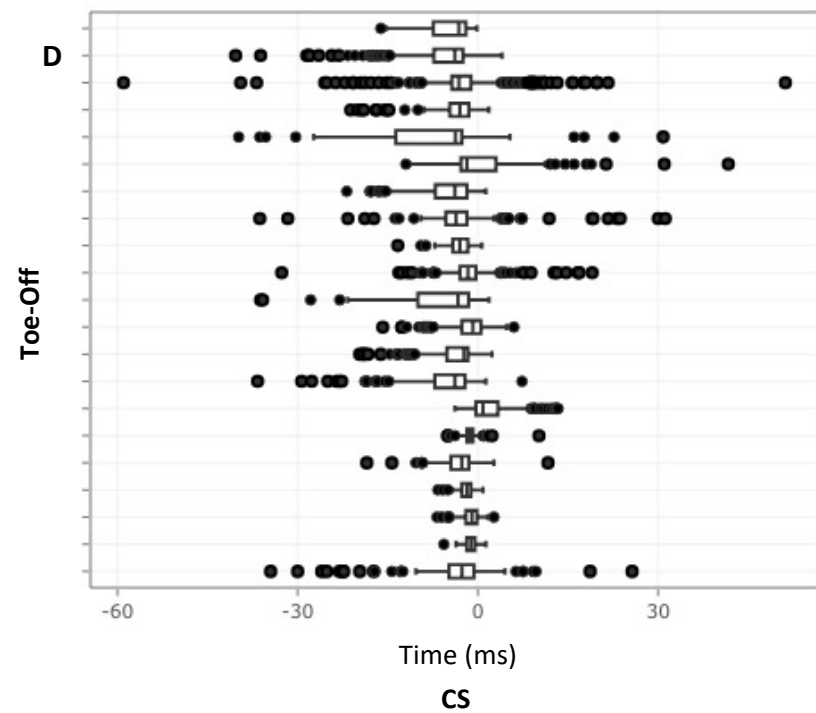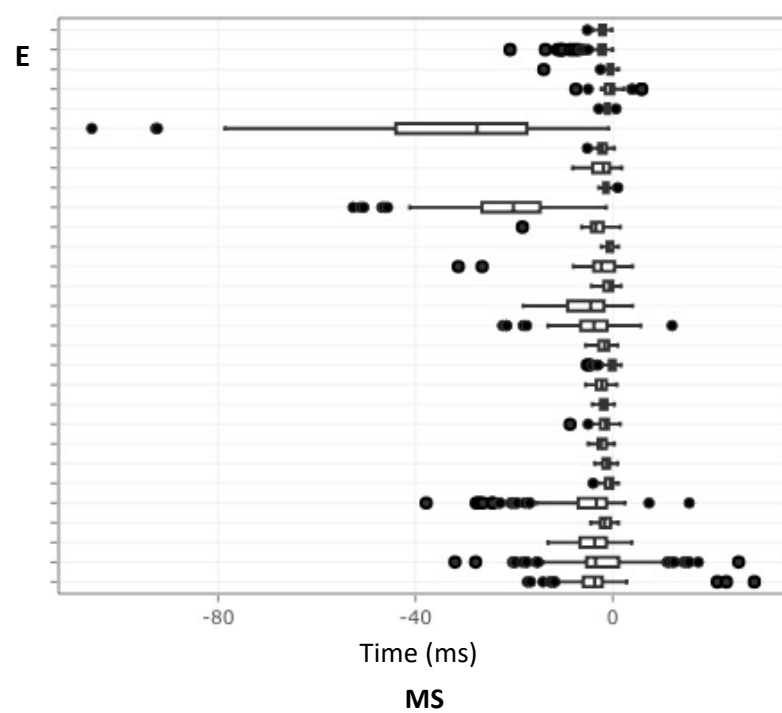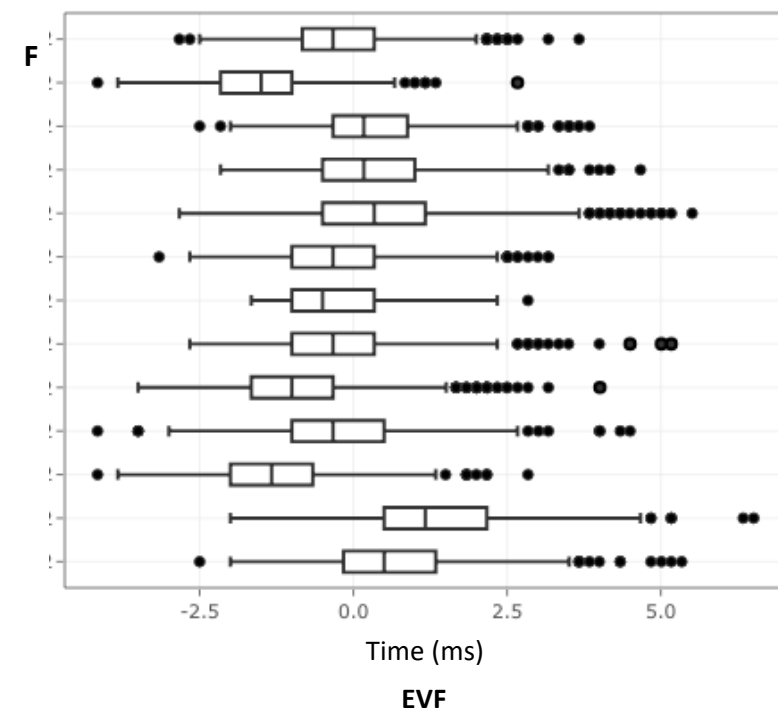

Supplement: Supplementary file 1 — Supplementary Material 1. [file 12984_2024_1405_MOESM1_ESM.pdf]
